# Weight management in obese pets: the tailoring concept and how it can improve results

**DOI:** 10.1186/s13028-016-0238-z

**Published:** 2016-10-20

**Authors:** Alexander J. German

**Affiliations:** Institute of Ageing and Chronic Disease, University of Liverpool, Leahurst Campus, Chester High Road, Neston, Wirral CH64 7TE UK

**Keywords:** Overweight, Dog, Cat, Dietary energy restriction, Success, Rebound

## Abstract

Obesity is now recognised as the most important medical disease in pets worldwide. All current strategies for weight management involve dietary energy restriction with a purpose-formulated diet. Whilst current weight management regimes can be successful, outcomes are often disappointing with the rate of weight loss progressively slowing down as time goes on. Success is most challenging for the most obese dogs and cats that are more likely to discontinue the programme before reaching target weight. To improve outcomes, clinicians must focus carefully on better tailoring programmes, paying particular to setting an appropriate target weight so as to maximise the benefits for the individual. In this opinionated review, the author will discuss findings from recent clinical research studies examining weight management in obese dogs and cats. A strategy for tailoring weight management targets will then be discussed, illustrated with case examples.

**Electronic supplementary material:**

The online version of this article (doi:10.1186/s13028-016-0238-z) contains supplementary material, which is available to authorized users.

## The significance of obesity in pets

In humans, it is now agreed that obesity is a disease [1], which can predispose to the development of various other diseases (e.g. cardiovascular disease, diabetes mellitus, liver disease, respiratory disease, neoplasia, and osteoarthritis) [2–5], and increase mortality risk [6]. Most veterinarians would agree that obesity is also an important medical disease in dogs and cats [7]. Overweight cats are at increased risk of developing diabetes mellitus, neoplasia, skin disease, oral cavity disease and urinary disease [8], whilst overweight dogs can suffer from diabetes mellitus, osteoarthritis, and urinary incontinence [9]. Increased adiposity can also adversely affect respiratory function [10, 11], cause metabolic derangements including insulin resistance [12–15], and also affect renal function and health [16]. Further, dogs that are fed ad libitum are more likely to be overweight, leading to a foreshortened lifespan [17]. Therefore, a wealth of information is now available to demonstrate the adverse health effects that can arise when pet dogs and cats become overweight. However, in light of recent evidence demonstrating that obese dogs have a poorer quality of life [18], the condition also presents a major welfare challenge for veterinary surgeons.

## Overview of obesity management

Successful weight management has two main phases, weight loss and subsequent weight maintenance. During the weight loss phase, it is essential that dietary energy intake is less than energy expenditure. In the last 10 years, two licensed drugs, dirlotapide and mitratapide, were available for the weight loss phase in dogs, both of which primarily acted by reducing appetite and thereby voluntary intake [19, 20]. Despite encouraging results from clinical trials, neither drug received widespread acceptance in clinical practice and, consequently, both have been withdrawn [21, 22]. Therefore, current weight loss strategies involve dietary energy restriction using a purpose-formulated diet, usually in combination with increasing activity and thereby expenditure [23–27]. Most weight loss diets are less energy dense, with increased amounts of good quality protein and micronutrients so as to reduce the risk of nutrient deficiencies developing and preserving lean tissue mass when food is restricted. For cats, feeding a diet containing 40 % of crude protein (on an as fed basis) during weight loss results in greater loss of fat mass and greater retention of lean body tissue than feeding a diet containing 30 % crude protein [28]. Similar results demonstrating preservation of lean mass during weight loss in obese dogs when comparing high protein (48 % as fed) and moderate protein (24 % as fed) diets [29].

The most effective diets also aim to minimise signs of hunger, in order to reduce begging behaviour and improve compliance, and this is usually achieved by altering macronutrient content [30, 31]. In dogs, supplementing both protein and fibre (e.g. 32 % crude protein and 28 % total dietary fibre, as fed) in a diet is best at reducing voluntary food intake [30], and improving outcomes of weight loss [32]. The protein and fibre content of a feline weight loss diet, must be carefully controlled, so as to inhibit hunger whilst ensuring palatability and, at the same time, preserving lean body mass. Although supplementing dietary fibre decreases voluntary food intake in cats, diets become less palatable if too much fibre diets is added [33]. Further, increasing protein intake generally increases, rather than decreases voluntary food intake [33]. Therefore, diets that minimise signs of hunger, whilst preserving lean tissue and not compromising palatability, have modestly increased dietary fibre (e.g. 23 % total dietary fibre as fed) and protein (e.g. 34 % as fed) [34].

## Success feels good: the benefits of weight loss in obese pets

A mistake many veterinarians make is to focus too heavily on the ‘mechanics’ of a weight loss programme (i.e. calculating energy intake, diet type, rate of weight loss, estimating target weight), and pay insufficient attention to the overall aim, which is to improve the pet’s quality of life, permanently. Thus, rather than worrying if weight loss is too slow, or whether the animal will ever reach its ‘perfect weight’, the clinician could focus on whether the animal is happier, more active, and less affected by any concurrent disease it may have (e.g. lameness, diabetes mellitus, respiratory diseases etc.). Further, success should be viewed less in terms of reaching a nominal ‘perfect weight’, than in permanently maintaining any weight lost, so that the health benefits of weight management are maintained. Unless bad owner habits are permanently changed (i.e. avoiding excessive treating, insufficiently exercising their pet), weight management will fail in the long run.

As described above, obesity can affect health in various ways, for instance by decreasing longevity [17], increasing the risk of other diseases developing [8, 9]. Therefore, in theory at least, a range of benefits might be expected for dogs and cats that lose weight successfully, for example longer lifespan, decreased risk of developing new diseases, improved organ function, and improvement in signs of any pre-existing disease. However, as of yet, no published studies have demonstrated that weight loss can either prevent diseases from developing or increase lifespan, possibly because such studies are difficult to perform. That said, there is good evidence that weight loss improves respiratory function [11], insulin sensitivity and other metabolic derangements [12–16], and also improves mobility in obese dogs with concurrent osteoarthritis [35, 36]. Most strikingly, force-plate analysis has demonstrated that mobility benefits begin very quickly during the weight loss process, e.g. once more than 6 % of body weight has been lost [36]. This modest amount of weight loss can readily be achieved in over 80 % of dogs that start a weight loss programme [37].

In addition to health benefits, success of weight loss should also be judged in terms of the impact on wellbeing. Recently, health-related quality of life was assessed in obese dogs before and after weight loss using a validated questionnaire [18]. When the dogs were obese, quality of life was worse than a group of control dogs in optimal body condition. However, quality of life had improved significantly after successful weight loss, with activity-related improvements being most marked. Such improvements were positively correlated with the amount of body fat lost. In summary, although there are many theoretical benefits to weight loss, those that have been clinically proven to date involve benefits in organ function, decreased severity of clinical signs from concurrent diseases, and improvements in quality of life.

## Weight loss is tough: ‘real world’ outcomes of weight loss in obese pets

Most studies that have assessed weight loss in obese dogs and cats have used colony animals, and protocols are invariably highly successful [19, 23, 27]. Typically, young, healthy dogs and cats are used that are often only modestly (e.g. ≤20 %) overweight. In such a setting, weight loss is predictable, usually progressing at 1–2 %/week, when energy intake is restricted to 50–75 % of maintenance energy requirements [23, 27, 29, 38]. Given such results, the vast majority of weight loss studies in colony was completed in 3–6 months. Whilst there are many advantages to undertaking colony research, the main disadvantage is that the findings cannot easily be generalised to weight loss in obese pets, where weight programmes rely on compliance from owners. It is only by examining the weight loss in clinical studies involving pet animals, that the outcomes of weight loss can be appropriately judged.

In light of this, recent studies have examined a period of weight loss in pet dogs and cats that are overweight. In both species, more marked energy restriction is usually required than in a colony and, despite this, rates of weight loss [0.5–1.0 % of starting bodyweight (SBW) per week in both species] are slower [24–26, 32, 34]. A number of factors account for the differences, such as the fact that most obese pet dogs and cats are neutered, have a variable age range, are more overweight, and commonly have concurrent disease [25]. However, the role of the owner is arguably most critical, most notably through poor compliance with veterinary advice. Indeed, self-reports of both dog and cat owners confirm that feeding extra food (such as table scraps and treats) is common, and the weight management food is not reduced to compensate [25, 26]. This suggests that, in contrast to the findings from colony studies, weight loss in obese pets is extremely challenging, especially for owners.

## Failure of weight loss programmes is common in obese dogs and cats

A number of studies have now assessed weight loss protocols in a clinical setting, but very few have examined success in terms of completion rates. This is because the majority of recent studies are short-term, typically assessing the first 2–3 months of weight loss [24, 27, 34]. Whilst these results are more representative than colony studies, and can demonstrate how successful the early stages of weight loss can be, they do not reflect performance of a complete weight loss cycle, especially in reaching target weight.

One study in dogs examined success of weight loss in terms of completing a programme [24]. Disappointingly, only 32/60 (53 %) completed the 6-month programme, and providing additional education for owners (in terms of classes discussing nutrition-related topics) did not improve compliance. A more recent study has also examined compliance with weight loss protocols in dogs [39]. A total of 143 dogs were studied, all referred to a specialist clinic for weight management, and 87 of these dogs successfully reached their target weight. There were various reasons why the remaining dogs stopped their programme prematurely: 11 (8 %) were euthanased for reasons unrelated to the obesity or stopped because another disease developed (1 dog); in other cases, the owners decided to stop, often because they had second thoughts immediately after enrolment (5 dogs, 3 %), they had struggled to comply with the requirements of the programme (3 dogs, 2 %) or for personal reasons (9, 6 %; including family illness, bereavement, and separation. The reason why the remaining 20 owners (14 %) chose to stop was unclear since they repeatedly refused to respond to any contact from veterinary staff. Multiple regression analysis demonstrated that the main factors that had the greatest effect on success were the rate of weight loss, the degree of obesity, and the type of diet fed. In this respect, rate of weight loss was faster in dogs that completed their programme than in those that stopped. With regard to degree of obesity, the fatter the dog, the less likely it was to complete, with a 15 % increase in the odds of failure for every additional percentage of excess body fat. Finally, a number of weight and dry weight loss diets were used in the study, and owner preferences were taken into account when selecting the particular diet to use. An additional study observation was that, when other factors were taken into account, dogs that had been fed dry food exclusively were significantly more likely to reach their target weight than dogs fed either wet food or a mixture wet and dry food [39].

In addition to the effect of the type of diet used during weight loss, feeding practices before weight loss could also be considered. In another study, possible effects of main meal feeding (type of food, how measured, meal frequency) and the feeding of extras (table scraps or treats) on the success of weight loss programme was assessed [40]. Most feeding practices had no significant effect; however, dietary energy intake during weight loss was less for dogs that were fed purchased snacks before weight loss, most likely because they continued to use these during the weight loss period. A final possible factor in compliance with a weight loss plan could be financial concerns. That said, cost of multiple visits to the veterinarian is unlikely to be a factor because, at least in the UK, these are usually offered free of charge provided that owners pay for the therapeutic weight loss food. Given that such diets are more expensive than standard maintenance diets, cost concerns could be a reason for an owner discontinuing if progress is slow. However, in a recent study, the average daily feeding cost during weight loss in dogs was not significantly different from the daily feeding cost before weight loss [41]. This implies that, for most dogs, the weight management regime is cost-neutral.

In a further recent study, outcomes of weight loss were assessed in cats, which demonstrated similar challenges in this species, with only 28 of 62 cats (45 %) successfully reaching target weight [42]. Again, the degree of obesity was the strongest predictor of failure (fattest cats more likely to fail). Whilst these findings may be disappointing, 85 % of pet dogs and 80 % of pet cats lose more than 6 % weight which, as stated above, would be sufficient to produce measurable health benefits [37, 42].

Therefore, current evidence suggests that approximately half of obese pets that start a weight loss protocol successfully reach their target weight. Perhaps even more disappointing is that fact that many animals subsequently regain weight. For example, in one recent study examining long-term follow up after weight loss protocol in obese dogs, 16 of 42 (48 %) regained some weight [43]. The main risk factor for regain was if the diet was if owners switched back to a standard adult maintenance food during weight loss, rather than continuing with the purpose-formulated weight management diet. In a separate canine study, faster rates weight loss led to more rapid rebound subsequently [38]. Rebound has also been demonstrated in after weight loss in cats, with 12 of 26 cats (46 %) regaining some weight [44]. These studies highlight the fact that weight management is a lifelong process with clinicians needing to continue to monitor body weight after ideal weight has been achieved.

## The law of diminishing returns

As discussed above, weight loss can have many benefits, but the process is challenging, and failure is common. However, a number of studies have suggested that outcomes vary depending upon the stage of the weight management process. Three examples will be discussed, namely changes in body composition, rate and energy intake during weight loss, and overall compliance.

### Changes in body composition during weight loss

Most studies assessing changes in body composition during weight loss in dogs and cats have been undertaken in a colony setting, and often suggest that lean mass is preserved [27, 45]. However, as discussed above, these studies generally examine modest amounts of weight loss (e.g. <20 %), and pets are usually more markedly obese. When a complete weight loss cycle is examined (i.e. restoring ideal weight) in obese pet animals, significant lean tissue loss is seen [25, 26, 32, 39]. The reason such results differ from colony studies is most likely due to the greater degrees of weight loss that are needed. Various factors are associated with lean tissue loss in obese animals during weight management, including rate of weight loss [46], decreased plasma adiponectin [15], and increased plasma clusterin [16]. However, the factor that appears to have greatest influence on how much lean tissue is lost is the overall amount of weight lost [25, 26]. These studies demonstrate that significant lean tissue loss only really occurs when dogs lose more than 20 % of their starting body weight.

### Rate of weight loss and energy intake during a complete weight loss protocol

Recent studies in both dogs and cats have examined rates and energy intake at different stages of a weight loss protocol reported a range of outcomes at key stages during a complete weight loss cycle [35, 39]. In the first 28 days of a weight loss protocol, the median rate of weight loss was 1.2 % body weight per week (BW/wk) in dogs [37] and 0.8 % BW/wk in cats [42]. Thereafter, weight loss rapidly slows in both species such that, after 12 months, median rate is <0.3 %/week in both species [37, 42]. This slowing down of the rate of weight loss occurs despite the energy intake being gradually reduced during the weight programme, by 10–20 % in both species.

### Compliance at different stages of a weight loss protocol

Compliance has also been assessed at different stages of the weight loss cycle in dogs and cats. Early on, compliance is good in both species, with 86 % of dogs and 82 % of cats remaining on the weight loss programme in the first 12 weeks. During this time, dogs and cats will have lost an average of 12 and 8 % of their starting body weight, respectively. Compliance progressively decreases so that, by 48 weeks, only 27 and 32 % of dogs and cats, respectively, are persisting with the programme.

### Summary

Based upon the published research, weight loss in obese pets is a clear example of diminishing returns, whereby the more weight that must be lost, the more difficult it is. Clinicians must accept the fact that failure is common, especially for the most obese animals. Whilst current weight loss strategies are not perfect, there are ways that veterinarians can get the best out of them, and ensure the greatest benefit to quality of life in patients under their care.

## Tailoring weight management protocols: the key to improving success

The research described above has emphasised a number of key aspects of current weight management strategies, which are summarised as follows:

### Successful weight loss has measurable health and welfare benefits when an obese dog loses weight

As described above, clinically proven benefits of successful weight loss include improved respiratory function [11], improved insulin sensitivity [12–15], improved mobility where there is concurrent orthopaedic disease [35, 36], and improved quality of life [18]. Other benefits are possible, including longer lifespan and disease prevention, although these have not yet been proven.

### Weight management protocols are difficult and many animals fail

In contrast to the results of colony studies, weight loss in obese pets is extremely challenging, requiring marked energy restriction. Rates of weight loss decline steadily over a weight loss cycle, which can be frustrating for the owner and may lead to poor compliance, leading to feeding of extra food or withdrawal.

### Lean tissue loss is common when obese animals lose significant amounts of weight loss

In the early stages of weight loss, lean tissue mass is preserved. Progressively increased lean tissue loss occurs once dogs and cats have lost more than 20 % of their body weight.

### Failure is common on current weight loss strategies

Approximately half of obese pet dogs and cats successfully reach their target weight during weight loss, and about half of these subsequently regain some weight. Compliance gradually worsens during a weight loss cycle in both dogs and cats, being good (>80 %) in early stages, but the rate of withdrawal gradually increases thereafter. Not surprisingly, dogs and cats with the most weight to lose are those that are the least likely to succeed.

### Most dogs lose some weight, even if they do not reach target

Although many dogs and cats are unsuccessful, the majority will lose some weight in the early stages, with at least 80 % losing >6 % weight, the cut-off for improvements in health in one study [36].

Faced with the current reality, clinicians should aim to maximise success by better *tailoring each weight management regime* to the individual animal. Case examples demonstrating how to tailor weight loss are provided in the online supporting information. Attention should be paid to considering what the key priorities for weight management are for the case, such that the most appropriate target weight is set which will maximise the benefit, whilst at the same time minimising the chance of failure. Two opposing strategies for setting target weight will be described, along with benefits and pitfalls. However, in reality, they represent opposite ends of a spectrum for decision making, which are highlighted by the case examples (Additional file 1: S1–S4).

### Complete weight loss

As the name suggests, the aim *complete weight loss* is to return the animal to its ideal weight, namely a weight whereby body composition is optimal. By reducing adipose tissue mass to this degree, the benefits to health are likely to be maximal. Indeed, the degree of improvement in quality of life is proportional to the amount of body fat lost in obese dogs. Although not yet proven, it might be that complete weight loss will extend lifespan and also prevent other diseases from developing. Such an approach would be most beneficial for young dogs, since their remaining lifespan is likely to be long, effects on organ function will have been short-lived, and associated diseases they are unlikely to have developed yet. Of course, the main disadvantage of attempting complete weight loss is the fact that the overall likelihood of failure is greater, and rebound would be expected.

### Partial weight loss

The philosophy of *partial weight loss* is for the clinician deliberately to set a target weight, which is above an animal’s ideal weight. Thus, rather than striving for the ‘perfect weight’, the aim is to achieve enough weight loss that quality of life is improved. As mentioned above, even very modest amounts of weight loss (e.g. ≥6 %) are sufficient to alleviate signs of concurrent disease such as osteoarthritis [36]. The main advantage is that such programmes can be relatively quick (2–3 months) and most animals are likely to succeed. The main disadvantages of such a programme is that patients are still likely to be overweight, so there may any benefits on lifespan or disease prevention will be less pronounced. For that reason, this strategy would mainly be used in older animals or for those who already have pre-existing diseases.

A final advantage of partial weight loss is the fact that, if the animals successfully reaches their target, and provided the owner remains committed, a second target weight could then be set. Thus, weight loss could progress in a series of stages, to a point whereby benefits have been maximised but not at the expense of owner compromise.

## Conclusions

Obesity is a major health concern both in pet cats and dogs, and successful weight management brings many benefits. However, the weight loss process is extremely challenging, especially for the most obese animals. Clinicians can get the best out of currently available weight loss strategies by better tailoring the weight loss programme to the individual, most notably by setting a realistic target weight. Returning an obese animal to its ideal weight is not always possible, and may not actually be essential to achieve the desired benefits to health and quality of life.

## Additional file

**Additional file 1.** Tailoring weight management in obese dogs—case examples.
