# Supplementary material for: Weight management in obese pets: the tailoring concept and how it can improve results
Source: Acta Vet Scand. 2016 Oct 20;58(Suppl 1):57. doi: 10.1186/s13028-016-0238-z (PMC5073926; doi:10.1186/s13028-016-0238-z)
Supplement: Supplementary file 1 — Additional file 1. Tailoring weight management in obese dogs—case examples. [file 13028_2016_238_MOESM1_ESM.zip › root/Case1.html]

# Tailoring weight management: CASE 1

## Signalment *1 year 7 month Female Corgi*

### Presenting complaints

- Obesity: estimated to be 40% above ideal weight
- No associated diseases

## Recommendation for weight loss

A weight management programme aiming to return the dog to its ideal body condition, and then maintaining it long term.

### Rationale

Given the early onset of obesity, this dog will have a lifelong risk of gaining weight. Thus, early recognition of obesity,
and lifelong weight management is critical in this case. Returning such a case to optimal body condition (BCS 4-5/9), and maintaining this, will maximise the
likelihood of positive effects on longevity, and reduce the chance of obesity-associated diseases arising in the future.

### Outcome

Body weight before weight loss was 18.4 kg. A weight management programme was introduced, and body weight decreased to 11.0 kg, over 202 days,
equivalent to 40% body weight change. The weight was then stabilised and maintained for 4 years by undertaking regular weight checks during follow-up.

|  |  |
| --- | --- |
| Case 1 � before weight loss | Case 1 � after weight loss |
|  |  |
|  |  |
