# Supplementary material for: Weight management in obese pets: the tailoring concept and how it can improve results
Source: Acta Vet Scand. 2016 Oct 20;58(Suppl 1):57. doi: 10.1186/s13028-016-0238-z (PMC5073926; doi:10.1186/s13028-016-0238-z)
Supplement: Supplementary file 1 — Additional file 1. Tailoring weight management in obese dogs—case examples. [file 13028_2016_238_MOESM1_ESM.zip › root/Case2.html]

# Tailoring weight management: CASE 2

## Signalment *6-year-oldneutered female bulldog*

### Presenting complaints

- Obesity, estimated at ~35% overweight
- Grade II mast cell tumour
- Brachycephalic airway disease

### Recommendation for weight loss

Partial weight loss regime, initially aiming for ~10% of body weight loss.

### Rationale

Prognosis is guarded given the mast cell tumour, so it is unlikely that weight loss extend lifespan dramatically.
However, the obesity is adversely affecting respiratory function, exacerbating the existing airway disease and affecting the dog�s quality of life.
A modest amount of weight loss would have the potential to improve respiratory function, and improve wellbeing for the short term. However,
the degree of weight loss should be carefully tailored, and lean tissue loss should be avoided since this could exacerbate the effects of cancer cachexia.
Aiming for ~10% weight loss should be relatively easy to achieve, and lean tissue is likely to be preserved. Of course, patient welfare is key;
if the weight loss process is tough and difficult for dog or owner (e.g. excessive beginning), it might be sensible to abort the process altogether.

### Outcome

Body weight before weight loss was 18.4 kg. A weight management programme was introduced, and body weight decreased to 17.3 kg, over 47 days,
representing 6% body weight change. Chemotherapy for the mast cell tumour was provided during this time. Whilst respitatory function improved,
mast cell tumour progressed rapidly, and the dog was euthanased after a further 3 months.

## Case 2 � before weight loss
