# Supplementary material for: Weight management in obese pets: the tailoring concept and how it can improve results
Source: Acta Vet Scand. 2016 Oct 20;58(Suppl 1):57. doi: 10.1186/s13028-016-0238-z (PMC5073926; doi:10.1186/s13028-016-0238-z)
Supplement: Supplementary file 1 — Additional file 1. Tailoring weight management in obese dogs—case examples. [file 13028_2016_238_MOESM1_ESM.zip › root/Case3.html]

# Tailoring weight management: CASE 3

## Signalment *9 year 7month Neutered male Labrador retriever*

### Presenting complaints

- Obesity – estimated to be 40% above ideal weight.
- Severe multi-joint osteoarthritis, that is

## Recommendation for weight loss

Partial weight loss regime, initially aiming for ~10% of body weight loss; if successful, further targets could be set aiming for greater weight loss.

### Rationale

Given the dog’s age, it is unlikely that returning the dog to its ideal condition will extend lifespan dramatically.
The dog also already has severe, co-existing disease so, whilst other conditions might be prevented, this is less of a priority than for case 1.
Instead, the main priority for weight management should be to lessen the impact of existing diseases.
The initial target for weight loss of ~10% is recommended in light of the fact that this is sufficient to improve mobility,17,18 yet is far more realistic
than complete weight loss. Such a target is reached in at least 80% of cases within ~3 months. If progress is good at that stage, further weight loss
could then be considered, which will improve mobility and overall quality of life further.

### Outcome

Body weight before weight loss was 54.0 kg. A staged weight management programme was introduced, with the initial target being weight loss of 10%.
This was successfully achieved, and two further targets were then set (20% weight loss, then 30% weight loss).
Three separate surgical procedures (two hip replacements and one elbow replacement) were performed during the weight loss period,
with the first after 10% weight loss. In total, the weight loss regime lasted 270 days, and body weight decreased to 39.0 kg.
This represented a total weight loss of 29%. Although the dog never reached its ideal weight, mobility and quality of life were significantly improved.

|  |  |
| --- | --- |
| Case 3 – before weight loss | Case 3 – after weight loss |
|  |  |
|  |  |
