# Supplementary material for: Weight management in obese pets: the tailoring concept and how it can improve results
Source: Acta Vet Scand. 2016 Oct 20;58(Suppl 1):57. doi: 10.1186/s13028-016-0238-z (PMC5073926; doi:10.1186/s13028-016-0238-z)
Supplement: Supplementary file 1 — Additional file 1. Tailoring weight management in obese dogs—case examples. [file 13028_2016_238_MOESM1_ESM.zip › root/Index.html]

## TAILORING WEIGHT MANAGEMENT IN OBESE DOGS - CASE EXAMPLES

- Case 1: A 1y7m neutered female corgi, with no associated diseases
- Case 2: A 6y neutered female bulldog with a grade II mast cell tumour and brachycephalic airway
  disease
- Case 3: A 9y neutered male Labrador retriever with severe multi-joint osteoarthritis
