# Supplementary figures and images for: Weight management in obese pets: the tailoring concept and how it can improve results
Source: Acta Vet Scand. 2016 Oct 20;58(Suppl 1):57. doi: 10.1186/s13028-016-0238-z (PMC5073926; doi:10.1186/s13028-016-0238-z)

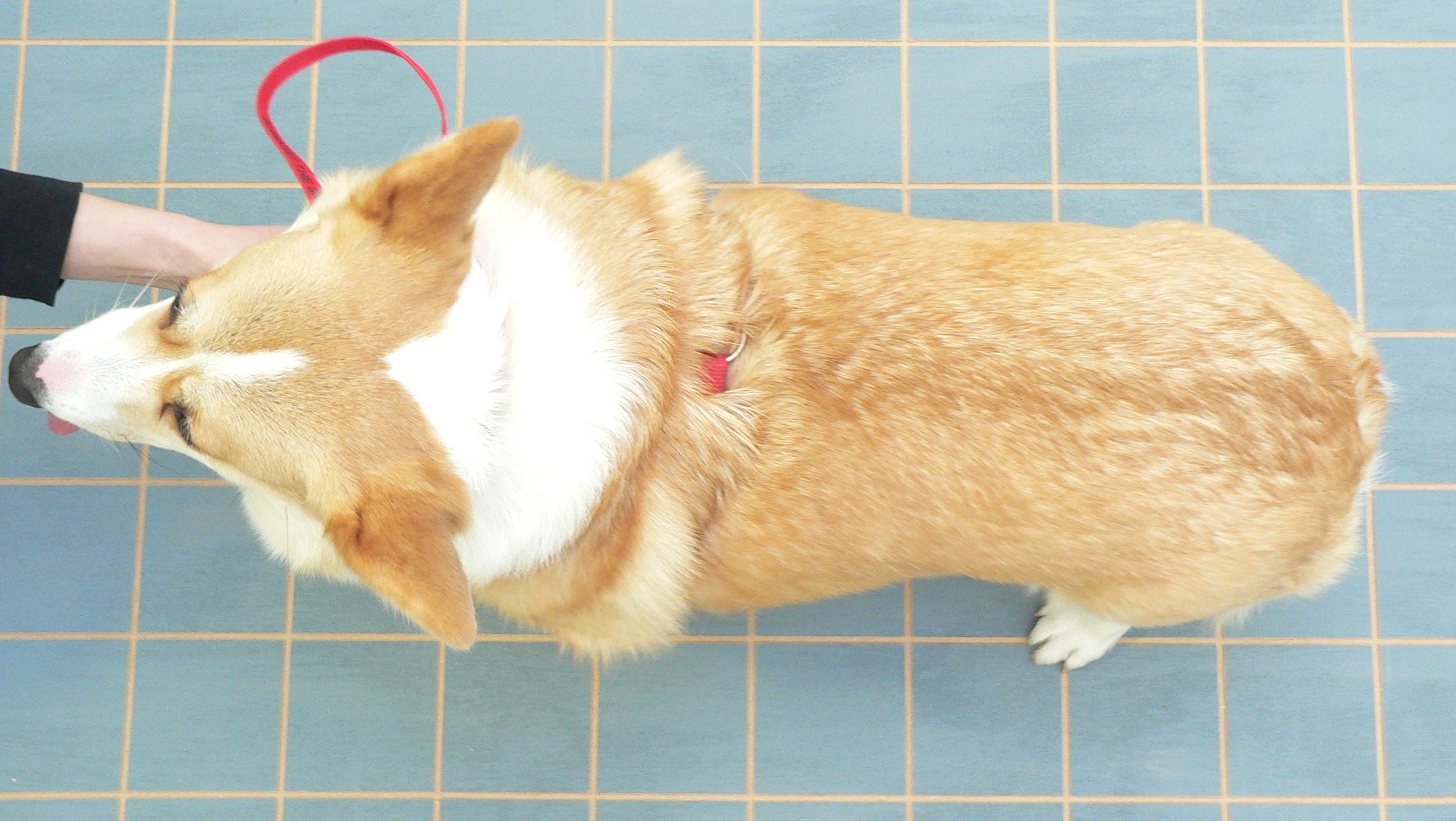

Supplement: Supplementary file 1 — Additional file 1. Tailoring weight management in obese dogs—case examples. [file 13028_2016_238_MOESM1_ESM.zip › root/images/1.jpg]

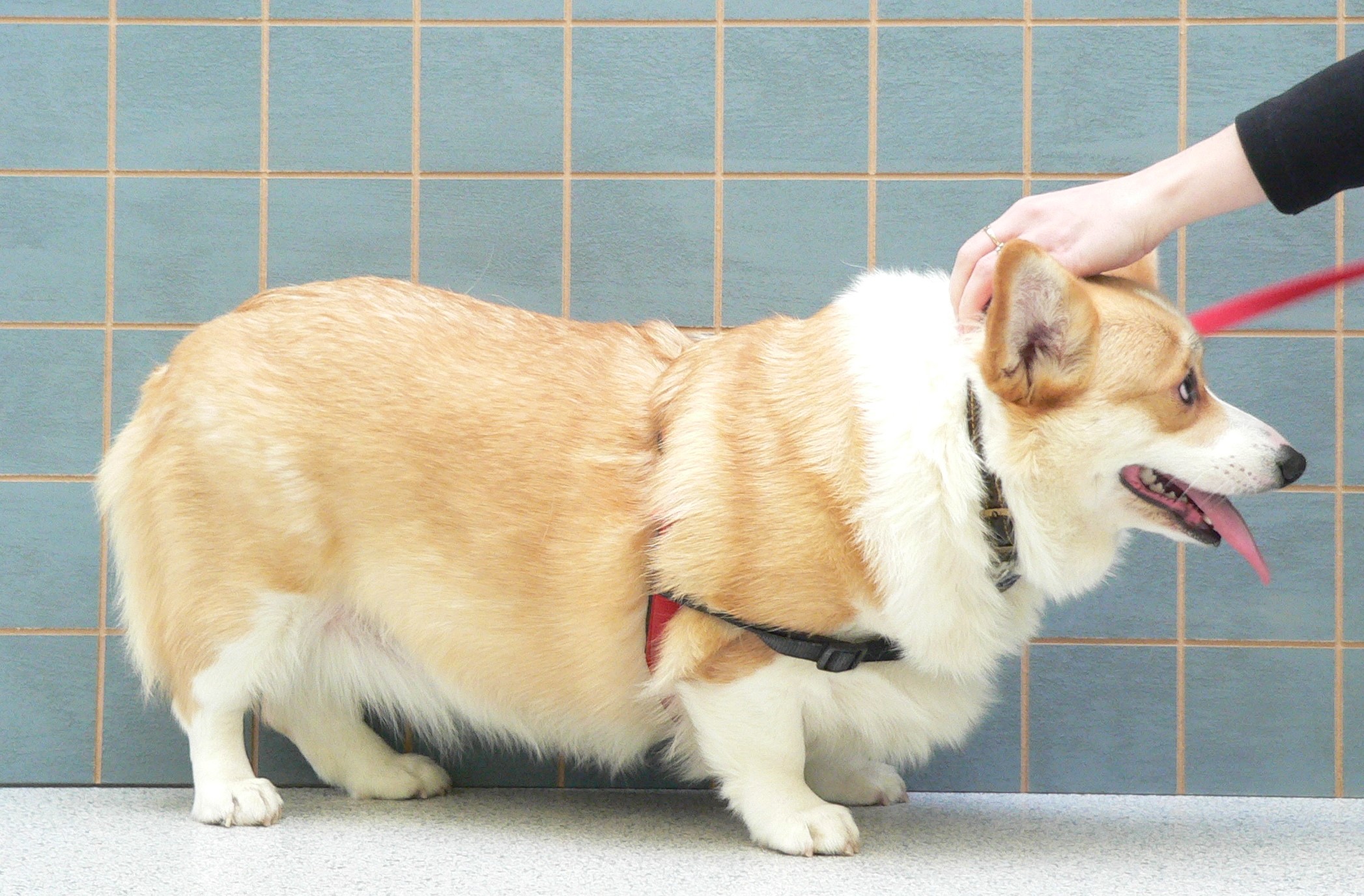

Supplement: Supplementary file 1 — Additional file 1. Tailoring weight management in obese dogs—case examples. [file 13028_2016_238_MOESM1_ESM.zip › root/images/2.jpg]

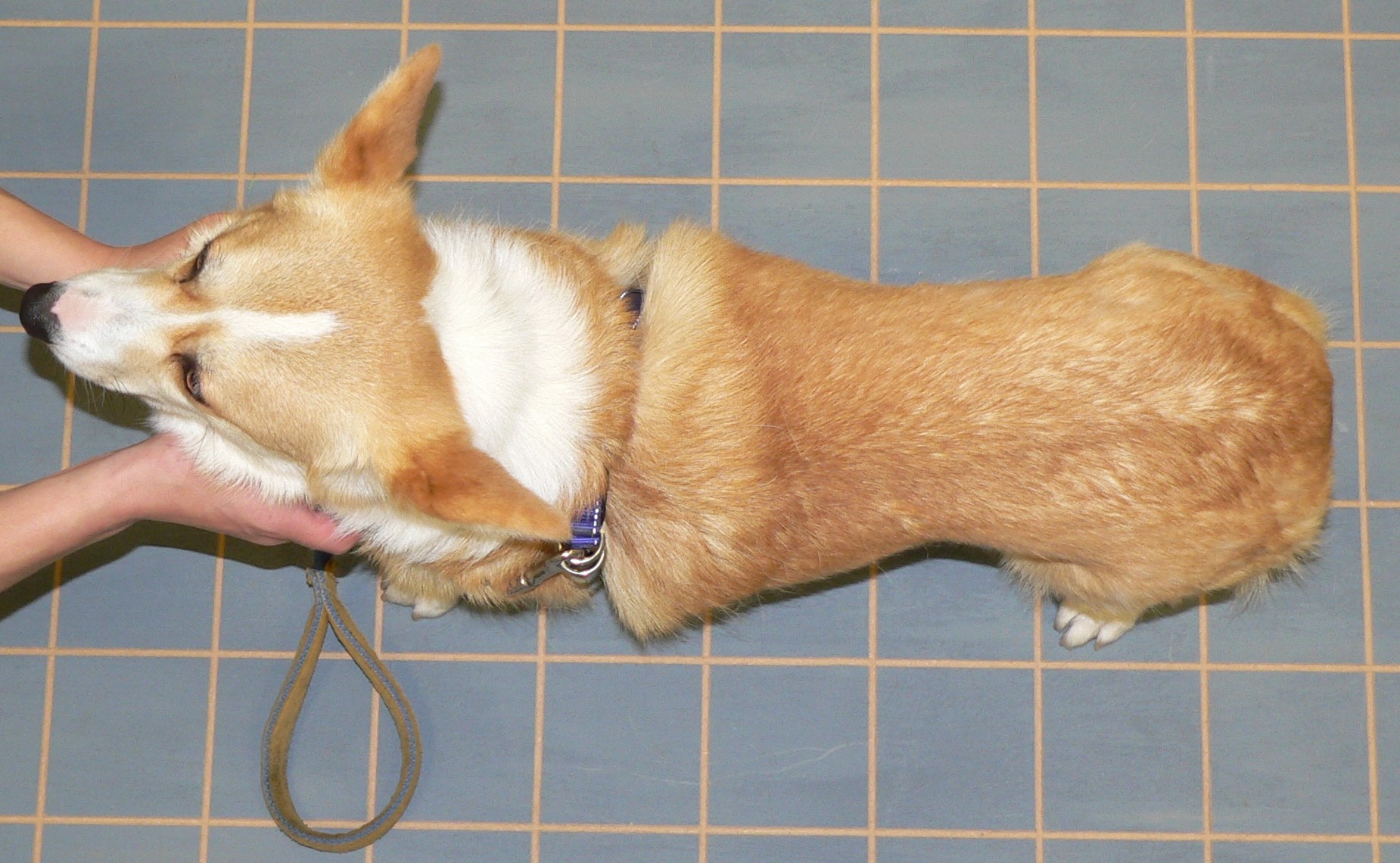

Supplement: Supplementary file 1 — Additional file 1. Tailoring weight management in obese dogs—case examples. [file 13028_2016_238_MOESM1_ESM.zip › root/images/3.jpg]

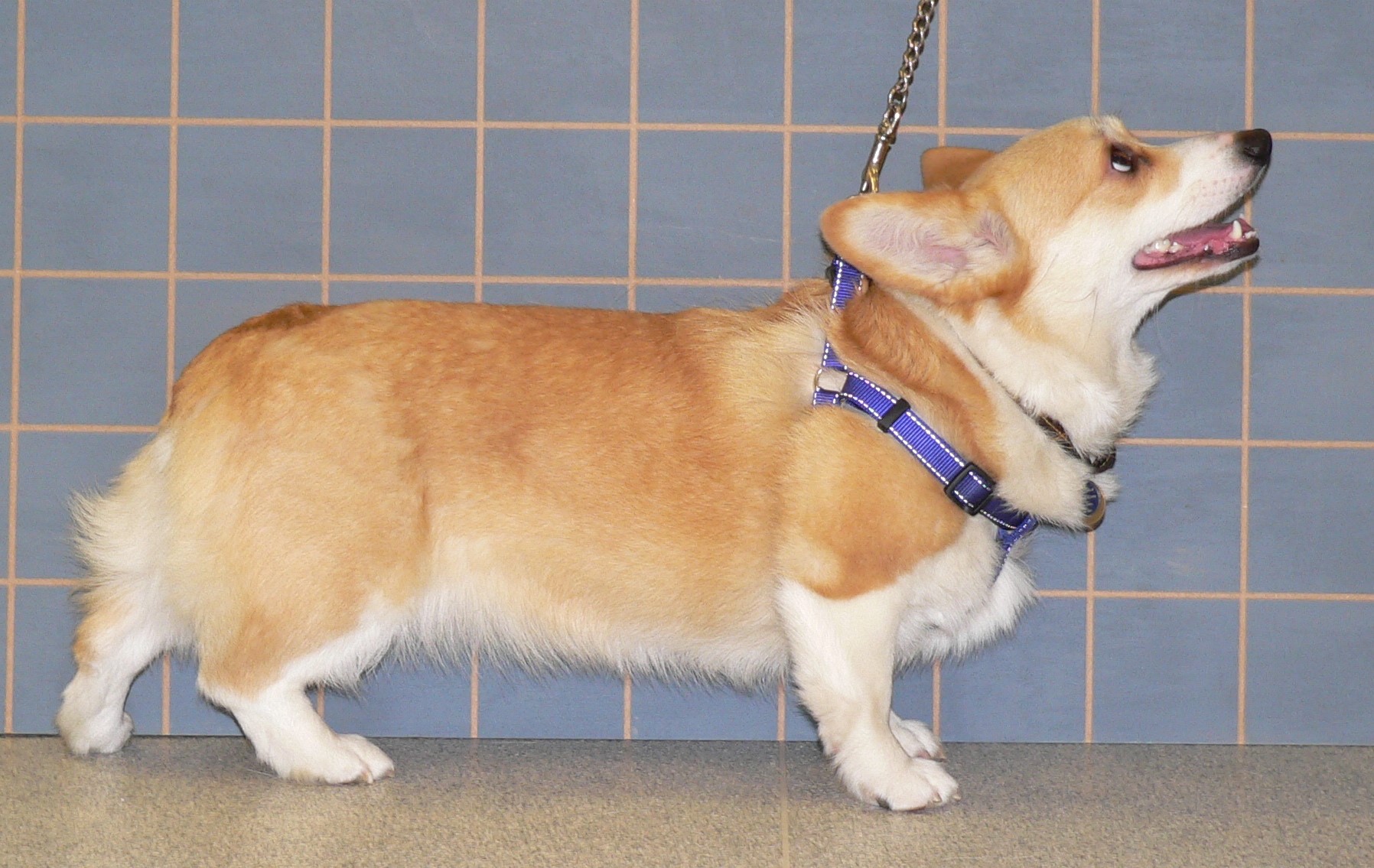

Supplement: Supplementary file 1 — Additional file 1. Tailoring weight management in obese dogs—case examples. [file 13028_2016_238_MOESM1_ESM.zip › root/images/4.jpg]

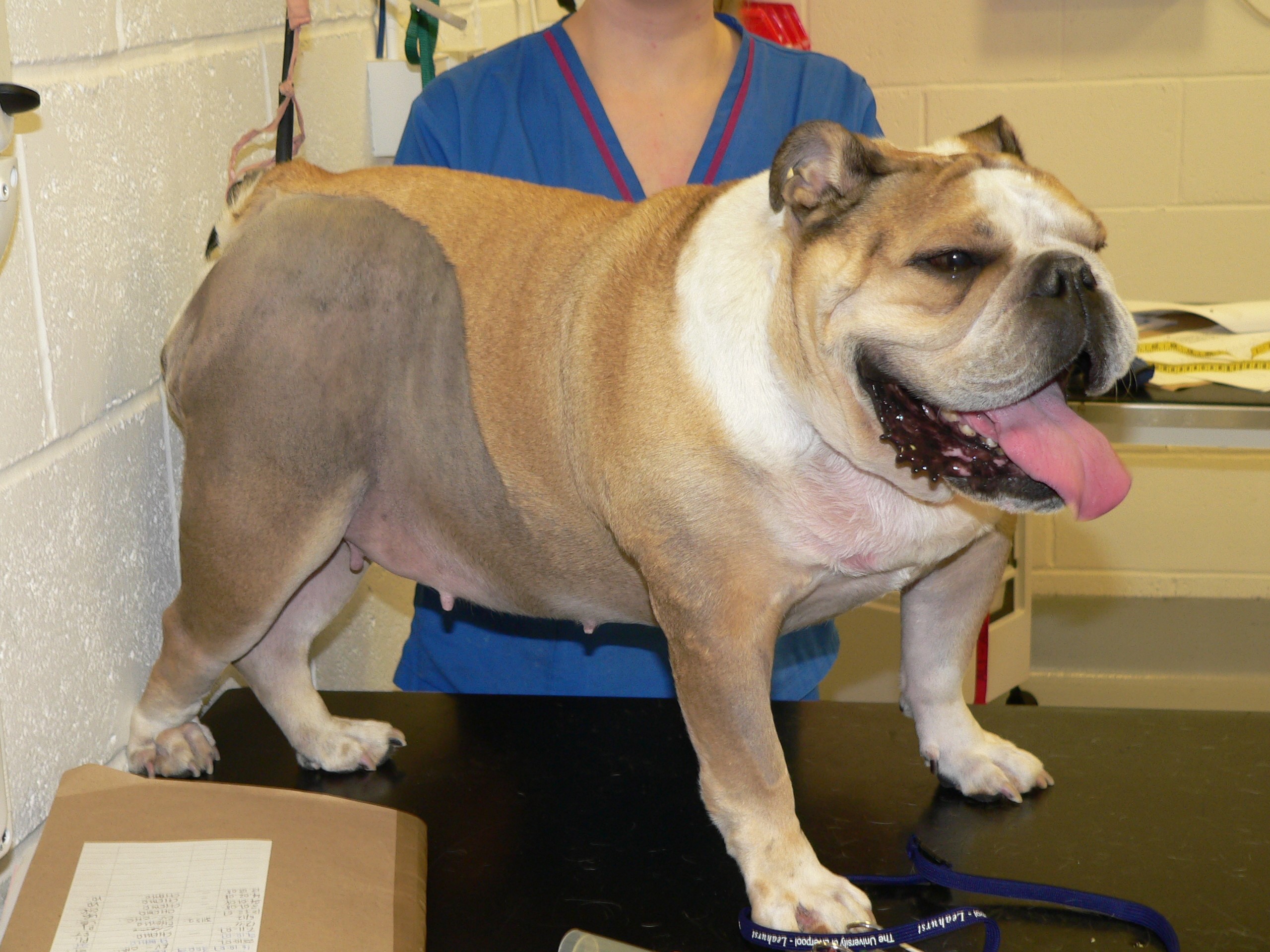

Supplement: Supplementary file 1 — Additional file 1. Tailoring weight management in obese dogs—case examples. [file 13028_2016_238_MOESM1_ESM.zip › root/images/5.jpg]

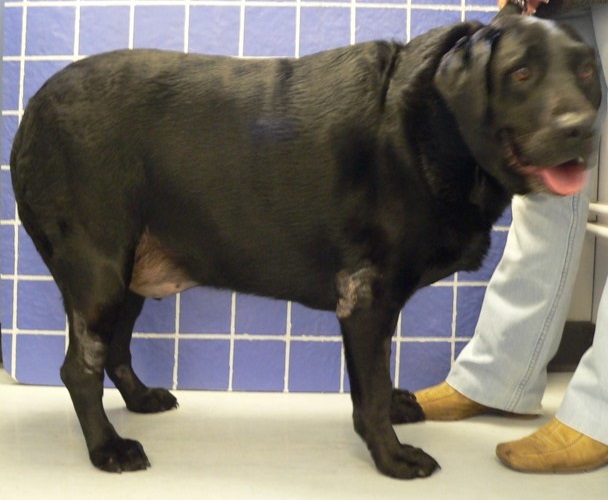

Supplement: Supplementary file 1 — Additional file 1. Tailoring weight management in obese dogs—case examples. [file 13028_2016_238_MOESM1_ESM.zip › root/images/6.jpg]

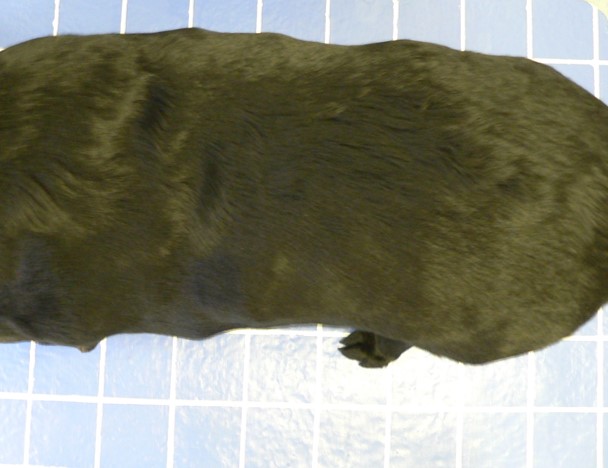

Supplement: Supplementary file 1 — Additional file 1. Tailoring weight management in obese dogs—case examples. [file 13028_2016_238_MOESM1_ESM.zip › root/images/7.jpg]

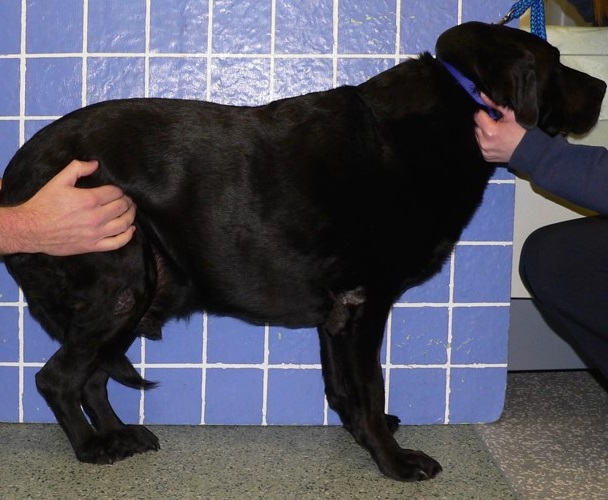

Supplement: Supplementary file 1 — Additional file 1. Tailoring weight management in obese dogs—case examples. [file 13028_2016_238_MOESM1_ESM.zip › root/images/8.jpg]

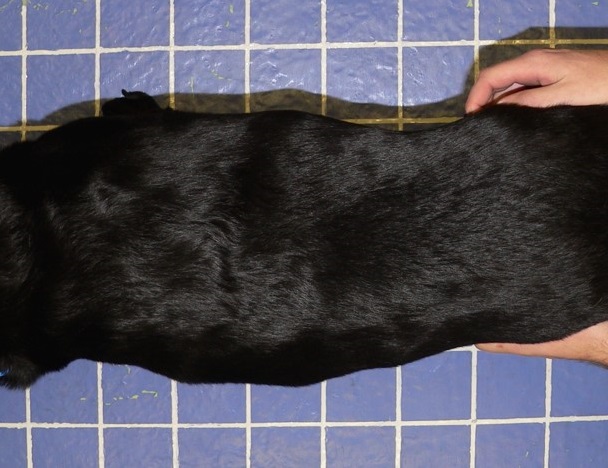

Supplement: Supplementary file 1 — Additional file 1. Tailoring weight management in obese dogs—case examples. [file 13028_2016_238_MOESM1_ESM.zip › root/images/9.jpg]
